# The Role of Poly(ADP-ribose) Polymerase-1 in Rheumatoid Arthritis

**DOI:** 10.1155/2015/837250

**Published:** 2015-03-03

**Authors:** Samuel García, Carmen Conde

**Affiliations:** ^1^Laboratory of Translational Immunology and Department of Rheumatology and Clinical Immunology, University Medical Center, Lundlaan 6, 3508 GA Utrecht, Netherlands; ^2^Laboratorio de Investigación 8, Instituto de Investigación Sanitaria (IDIS), Hospital Clínico Universitario de Santiago de Compostela (CHUS), SERGAS, Travesia da Choupana s/n, 15706 Santiago de Compostela, Spain

## Abstract

Poly(ADP-ribose) polymerase-1 (PARP-1) is a nuclear enzyme with a crucial role in the maintenance of genomic stability. In addition to the role of PARP-1 in DNA repair, multiple studies have also demonstrated its involvement in several inflammatory diseases, such as septic shock, asthma, atherosclerosis, and stroke, as well as in cancer. In these diseases, the pharmacological inhibition of PARP-1 has shown a beneficial effect, suggesting that PARP-1 regulates their inflammatory processes. In recent years, we have studied the role of PARP-1 in rheumatoid arthritis, as have other researchers, and the results have shown that PARP-1 has an important function in the development of this disease. This review summarizes current knowledge on the effects of PARP-1 in rheumatoid arthritis.

## 1. Rheumatoid Arthritis

Rheumatoid arthritis (RA) is an autoimmune disease that is characterized by chronic inflammation that affects the peripheral joints and leads to the progressive destruction of the cartilage and bone. RA has a prevalence of 0.5–1% in the population worldwide, and genetic and environmental factors have been implicated in its aetiology. The age of onset is between 35 and 50 years and it is more common in women than in men (a 3 : 1 ratio), suggesting that hormonal factors are related to the development of the disease. Approximately 30% of RA patients have extra-articular manifestations, which contribute to the morbidity and mortality of the disease. Furthermore, this disease leads to a reduction in life expectancy between 3 and 10 years [1–4].

In RA, the initiation of an immune response against unknown antigens leads to the infiltration of the immune cells, primarily the monocytes/macrophages and B and T cells in the affected joints, and also to the activation and proliferation of the stromal cells of the joints, the fibroblast-like synoviocytes (FLS). The activated immune cells and FLS release inflammatory mediators, such as cytokines, chemokines, growth factors, and prostanoids, that perpetuate the inflammatory process and promote the hyperplasia of the synovial membrane; they also release matrix metalloproteinases (MMPs) and aggrecanases that digest the extracellular matrix and articular structures. These mediators also contribute to the formation of new blood vessels from the existing vasculature (angiogenesis), which provide nutrients to the inflamed joint and allow the infiltration of the immune cells into the synovium, thereby perpetuating the inflammatory process [4–7].

The final consequences of these processes are the destruction of the cartilage and the erosion of bone, leading to joint deformity and disability.

Among the plethora of inflammatory mediators playing a role in RA, interleukin-1*β* (IL-1*β*), interleukin-6 (IL-6), and tumour necrosis factor-*α* (TNF-*α*) have been shown to be the most relevant cytokines in the pathology. These cytokines are related to different processes in RA, such as the induction of inflammatory mediators and MMP expression by the FLS, monocytes/macrophages, and T cells; the activation, proliferation, and differentiation of the T cells; the induction of the B cells' proliferation and antibody production and the osteoclast activation. Blocking these cytokines has shown therapeutic efficacy, and three of the current biological therapies used for the treatment of RA target these cytokines [8, 9].

### 1.1. Molecular Pathways in Rheumatoid Arthritis

Although there are multiple signal transduction pathways involved in RA, the mitogen activated protein kinase (MAPK) pathways play a primary role in the inflammatory processes of the disease [10, 11]. ERK, JNK, and p38 are expressed and activated in the synovial tissue of RA patients, and IL-1*β* and TNF-*α* induce ERK, p38, and JNK activation in RA FLS [12]. The key role of the MAPKs in RA pathogenesis has been demonstrated in different studies, in which the absence or inhibition of MAPKs has been shown to reduce the severity of several models of arthritis [13–16].

Numerous reports have also shown the activation of different transcription factors in the synovium of RA patients, such as NF-*κ*B, AP-1, IRFs, and FoxO, as well as STAT family members [17].

NF-*κ*B is activated in the synovial tissue from RA patients; both subunits p50 and p65 are located in the nuclei of the synovial macrophages, and the RA FLS and TNF-*α* and IL-1*β* induce a rapid NF-*κ*B translocation in the RA FLS. Moreover, pharmacological NF-*κ*B inhibition and genetic NF-*κ*B deletion or modification decrease the severity and bone erosion in different arthritis models in animals [18, 19].

AP-1 is another transcription factor that is involved primarily in the process of joint destruction by inducing the expression of MMPs, but it also elicits other roles in RA, such as the production of inflammatory mediators and the induction of T cell differentiation and osteoclast formation. The key role of AP-1 in RA has been shown in animal models, in which the deletion or pharmacological inhibition of AP-1 reduces both the severity of arthritis and the production of inflammatory cytokines and MMPs [17, 20].

### 1.2. Experimental Arthritis Models

Several experimental models have been used to characterize the mechanisms involved in the pathogenesis of arthritis and to test new therapeutic strategies. Such models include collagen-induced arthritis (CIA), collagen-antibody-induced arthritis (CAIA), adjuvant-induced arthritis (AIA), and spontaneous arthritis models such as TNF-transgenic mice and the K/BxN mice.

The CIA model shares many clinical and histological features with human RA [21]. Similar to RA, mice develop symmetric peripheral joint inflammation, synovitis, and cartilage and bone damage. Mice also develop hyper *γ*-globulinemia, antibodies to type-II collagen, and rheumatoid factor. In this model, DBA/1 mice with the MHC class-II I-A^q^ haplotype develop arthritis following the injection of type-II collagen in complete Freund's adjuvant (CFA). The initial injection is usually followed by an intraperitoneal collagen booster 21 days later. Following immunisation, mice develop polyarthritis that reaches its severity peak at about day 35 [21–23]. One of the advantages of this model is that it allows the study of two phases in the development of arthritis: the initial autoimmune response, in which collagen-specific T and B cells are produced, and the effector phase, consisting of joint inflammation, cartilage damage, and bone erosion. On the other side, a notable disadvantage of this model is the variable incidence and progression of diseases between different laboratories. The CIA model has been a valuable tool to identify the involvement of inflammatory cytokines, autoantibody responses, and T cells in arthritis and for the preclinical testing of new treatments for RA [23].

CAIA is a variant of CIA, in which arthritis is induced by intravenous injection of an arthritogenic cocktail of monoclonal anti-type-II collagen antibodies [24]. This model reflects only the effector phase of arthritis, which develops within 48 hours of antibody injection. Clinical and histopathological features of CAIA are similar to CIA, although the infiltrate is composed mainly of macrophages and neutrophils. In addition, the incidence of arthritis reaches 100% and is independent of the MHC class-II haplotype. These two characteristics make this model particularly useful for studying arthritis in genetically modified mice [24, 25].

The adjuvant-induced arthritis (AIA) model is elicited by intradermal injection of CFA at the base of the tail of rats [26]. AIA is characterized by the rapid onset and progression of joint inflammation with marked cartilage and bone resorption. It shares features of human RA including joint inflammation, cartilage damage, and bone erosion. However, AIA also affects the spine, the skin, the eyes, and the gastrointestinal and genitourinary tracts, which are not involved in human RA [22]. The main advantage of this model is its reproducibility, as 100% of the animals develop arthritis by 14 days after inoculation of CFA, and its main disadvantages are the absence of humoral component and the dissimilarities with human RA regarding tissue damage.

On the side of the spontaneous arthritis models, the first developed was the TNF transgenic mouse, which was reported by Keffer et al. in 1991 [27]. The mice overexpress human TNF (Tg197) and spontaneously develop an erosive chronic polyarthritis that closely mimics human RA, with synovial hyperplasia, pannus formation, cartilage destruction, and bone erosion. This model has established that TNF plays a fundamental role in the pathogenesis of RA and it has been very useful in the assessment of the anti-TNF treatment. The main limitation of this model is the absence of the early autoimmune phase of arthritis.

Another widely used spontaneous model is the K/BxN arthritis model, described by Kouskoff et al. [28]. In this model, a T cell receptor (TCR) transgene recognizes endogenous glucose-6-phosphate isomerase presented on the MHC class-II molecule I-A^g7^. This autoimmune reaction induces an early and rapidly progressive arthritis that is T and B cell dependent and similar to human RA. It is a very reproducible model in which robust arthritis is developed in 100% of the transgenic mice. This model allows also transferring the disease to a large variety of strains using serum from K/BxN mice, which contains anti-glucose-6-phosphate isomerase pathogenic antibodies. The advantages of this model are its reproducibility and the ability to separate the immunization and effector phases of arthritis [29]. Its main limitation is that the pathogenic autoantibodies of this model are absent in human RA. The K/BxN transfer model has led to reappreciating the role of the humoral response in RA pathogenesis.

## 2. Poly(ADP-ribose) Polymerase-1

Several studies using experimental arthritis models [30–37] or pharmacological and genetic inhibition of PARP-1 in FLS from RA patients [38] have revealed the involvement of this protein in the pathogenesis of arthritis and the potential therapeutic effects of PARP-1 inhibition.

PARP-1 (EC2.4.2.30) is a nuclear enzyme with a key role in the maintenance of genomic integrity. PARP-1 is a highly conserved protein of 113 KDa with a ubiquitous expression encoded by the* PARP-1* gene, located in the human 1q41-42 chromosome. PARP-1 has three primary domains: an amino- (N-) terminal DNA binding domain (DBD), an automodification domain, and a carboxy- (C-) terminal catalytic domain. PARP-1 is the foundation and the most abundant member of the PARP family, which includes 18 members. All PARP members have a characteristic conserved catalytic domain located in the C-terminal region. According to their functional domains and functions, the members of the PARP family can be divided into five groups: DNA-dependent PARPs, tankyrases, CCCH-type zinc-finger PARPs, macroPARPs, and other PARPs [39–41].

PARP-1 is the most important member, exhibiting poly(ADP-rybosil)ation activity; in fact, 80–85% of this activity is mediated by PARP-1. The remaining poly(ADP-rybosil)ation activity is mediated by other members of the family, such as PARP-2, PARP-3, PARP-4, and tankyrases 1 and 2. Poly(ADP-rybosil)ation is a protein postransductional modification essential to cellular processes, such as the regulation of DNA reparation, the maintenance of chromatin function and genomic stability, the regulation of transcription, cell cycle progression, and cell death [39, 41].

In the poly(ADP-rybosil)ation process, PARP cleaves the NAD^+^ in the nicotinamide and ADP-ribose to form long and branched (ADP-ribose) polymers (PAR). The PAR binds to the acceptor proteins (including PARP-1 itself) through ester bonds to the residues of carboxyl-*γ* of glutamic acid and regulates their enzymatic activity or macromolecular interactions with other proteins or DNA or RNA molecules [40, 42].

PARP-1 has a key role in the maintenance of genomic stability, and the absence or deficiency of  PARP-1 leads to defects in the repair of DNA breaks, an increase in homologous recombination, sister chromatic exchange, and micronuclei formation [43]. PARP-1 is also involved in the regulation of diverse DNA reparation pathways, such as the BER (Base Excision Repair), SSBR (Single Strand Break Repair), and DSBR (Double Strand Break Repair) pathways [41, 44, 45]. Moreover, PARP-1 has been related to DNA damage-induced cell death and apoptosis, both caspase dependent and caspase independent, and the results suggest that the role of PARP-1 in cell death depends on the cell type and the type of stimulus [45–50].

### 2.1. PARP-1 in Inflammation

In recent years, new roles of PARP-1 have been discovered, and one of the most important is the role of PARP-1 in inflammatory processes. Different works have reported that both the pharmacological inhibition of PARP and a deficiency of PARP-1 have a protective role in inflammatory diseases, such as LPS-induced septic shock, uveitis, colitis, streptozotocin-induced diabetes, asthma-related lung inflammation, and atherosclerosis [33, 51–55]. In these models, PARP inhibition showed an anti-inflammatory effect, due primarily to a reduction in the expression of inflammatory mediators, the impaired recruitment of inflammatory cells, the inflammation sites, and the reduction of necrotic cells in the inflamed areas.

There are two possible molecular mechanisms that have been proposed to explain this resistance. The first mechanism is related to the overactivation of the PARP-1 and the depletion of NAD^+^ and ATP. In the inflammatory processes, the oxygen and nitrogen free radicals that are released result in DNA damage that leads to the constant activation of PARP-1 and consequently to the depletion of the cellular levels of NAD^+^ and ATP. The depletion of the cellular NAD^+^ and ATP pools produces an irreversible cellular energetic failure and cell death by necrosis. The necrotic cells release their cellular content into the extracellular space, perpetuating the inflammatory processes [40, 45, 56].

The second mechanism is related to the role of PARP-1 as a transcriptional regulator and to the ability of PARP-1 to modulate the expression of proinflammatory genes, primarily the cytokines and chemokines. PARP-1 regulates genomic transcription through 2 independent processes: the regulation of the chromatin structure and the regulation of the activity of the transcription factors. In the first mechanism, PARP-1 mediates the poly(ADP-rybosil-)ation of the chromatin-associated protein-like histones, leading to the dissociation of the nucleosomes dissociation and the relaxation of the chromatin structure. The relaxation of the chromatin allows the access of proteins implicated in the transcription into the DNA and the subsequent genomic expression. PARP-1 also regulates, through PARP-1 enzymatic activity, the activation of different transcription factors, such as NF-*κ*B, AP-1, AP-2, YY-1, Oct-1, Stat-1, B-MYB, HIF-*α*, and SP-1. Moreover, PARP-1 also modulates the activation of other transcription factors, including Oct-1, YY-1, B-MYB, and AP-2, through direct protein-protein interactions [40, 45, 56].

Of special interest is the role of PARP-1 in the transcriptional activation of NF-*κ*B and AP-1, as both transcription factors are related to different pathologies. PARP-1 regulates the activation of both transcription factors, and PARP inhibition or PARP-1 deficiency reduces the transcriptional activity of NF-*κ*B and/or AP-1 in models of septic shock, inflammation, and ischemic-reperfusion, as well as in cancer and RA [51, 52, 57–59].

Beyond the modulation of the transcription factors' activity, PARP-1 has also been related to the regulation of the MAPK pathways, and it has been shown that PARP inhibition reduces the activation of ERK, JNK, and p38 in inflammatory as well as in ischemic and oxidative stress processes [60–63].

Therefore, the protective effect of PARP-1 inhibition in inflammatory models is due primarily to the reduction of the transcription factors and the activation of the signalling pathways, leading to the downregulation of the expression of the proinflammatory mediators.

## 3. PARP-1 in Rheumatoid Arthritis

The studies of the role of PARP-1 in experimental arthritis models (Table 1) started in the 1990s, using unspecific inhibitors such as nicotinamide (NA) and nicotinic acid amide (NAA). These studies showed that PARP inhibition, alone or in combination with the TNF-*α* inhibitor thalidomide, reduced the severity of two different models of arthritis, potassium peroxochromate-induced arthritis and the collagen-induced arthritis (CIA) [30, 31]. However, due to the high unspecificity of the inhibitors used, the protective role of PARP inhibition in arthritis could not be concluded in these studies.

In the late 1990s, the second generation of PARP inhibitors was developed (Table 1). These inhibitors, such as 6-iodo-5-amino-1, 2-benzopyrone (INH_2_BP), N-(6-oxo-5,6-dihydrophenanthridin-2-yl)-(N,N-dimethylamino)acetamide hydrochloride (PJ34), 3,4-dihydro-5-[4-1-(1-piperidinyl)butoxy]-1-(2H)-isoquinolinone (DPQ), and 4-amino-1, 8-naphthalimida (ANI), are based on the pharmacophore benzamide (the analogue of NA), and they are very specific and strong PARP inhibitors [32, 45].

Independent studies have shown that the PARP inhibitors INH_2_BP and PJ34 reduce the incidence and severity of the CIA model. Interestingly, the therapeutic effects of both PARP inhibitors were associated with a reduction in the oxygen- and nitrogen-derived free radicals and a decrease in the neutrophil infiltration into the joints [33, 34]. Gonzalez-Rey et al. [35] also investigated the impact of the PARP-1 inhibitor AIQ in experimental arthritis. AIQ reduced the incidence and severity of established collagen-induced arthritis, completely abrogating the joint swelling and destruction of the cartilage and bone. The therapeutic effect of AIQ was due to the reduction of the inflammatory response and to the reduced Th1-driven autoimmune response, demonstrating the role of PARP-1 in the two crucial processes of RA, the initiation of the immune response and the initiation and perpetuation of inflammation [35]. Interestingly, the PARP-1 inhibitors PJ34 and AIQ also showed a prophylactic effect, as a protective effect was observed when the inhibitors were administered after the onset of arthritis.

Our group investigated the impact of the selective suppression of PARP-1, using PARP-1 deficient mice, in a model of arthritis induced by anti-collagen antibodies (CAIA). We observed that the absence of PARP-1 reduced the severity of the disease, although the incidence was not affected. This reduction was due to the reduced joint expression of the cytokine IL-1*β* and the chemokine MCP-1 (Table 1). Indeed, we showed that it is likely that PARP-1 is the member of the PARP family that is involved in arthritic inflammation, as the reduction in the severity of arthritis was similar in the arthritic PARP-1 deficient mice regardless of whether they were treated with the PARP inhibitor DPQ [36].

The role of PARP in arthritis has also been analysed in other animal models, and comparable results have been found. Mazzon et al. [37] showed that PARP inhibition by GPI 6150 treatment significantly reduced paw edema in the acute and delayed phases of inflammation in the rat adjuvant-induced arthritis model.

**Figure 1:**
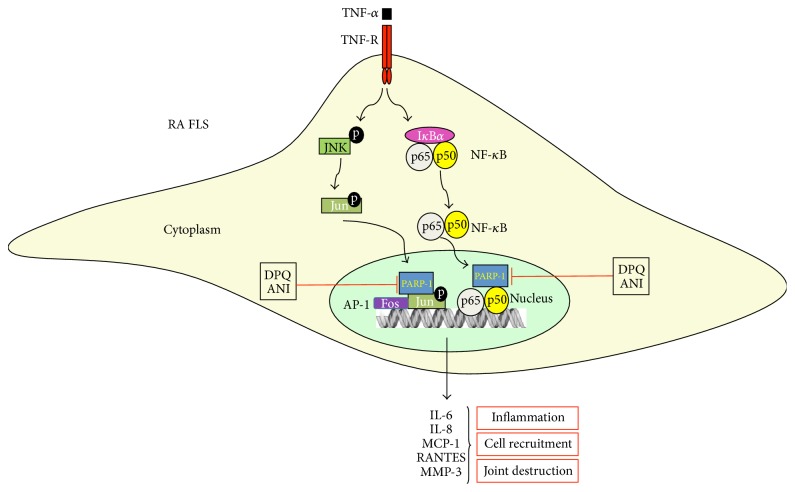
*Effect of PARP-1 on the TNF-α induced production of inflammatory mediators in RA FLS*. TNF-*α* stimulation induces the activation and the translocation to the nucleus of the transcription factors NF-*κ*B and AP-1. In the nucleus, PARP-1 enzymatic activity and/or direct protein interaction enhances the transcriptional activity of both NF-*κ*B and AP-1, leading to the production of inflammatory mediators.

To extend the knowledge about the role of PARP-1 in rheumatoid arthritis, our group analysed the effect of PARP inhibition on the cell proliferation, production of inflammatory mediators, and activation of molecular pathways in the RA FLS. The results showed that PARP-1 inhibition reduced the FLS proliferation and the TNF-induced IL-6, IL-8, MCP-1, RANTES, and MMP-3 production. PARP-1 suppression by siRNA confirmed the reduction of the production of the TNF-induced inflammatory mediators, suggesting that the reduced inflammatory response we observed was due to the inhibition of poly(ADP-ribosil)ation and not to effects other than the inhibition of the PARP function. Moreover, the results also suggest that PARP-1 is the PARP family member responsible for this reduction. Indeed, in this work we have suggested that the reductions in the production of inflammatory mediators and proliferation are most likely due to the reduced activation of NF-*κ*B and AP-1, as we observed a partially impaired NF-*κ*B and AP-1 binding activity after the treatment with the PARP inhibitors or after the siRNA PARP-1 transfection (Figure 1) [38].

Therefore, this work supports the previous results in animal models showing that PARP inhibition reduced the production of the inflammatory mediators involved in RA.

Taken as a whole, these studies suggest that PARP is involved in the progression of the inflammatory process of RA and that the pharmacological inhibition of PARP has an anti-inflammatory potential in arthritis diseases.

## 4. PARP-1 Inhibitors in Clinical Trials

Due to the crucial role of PARP-1 in the pathophysiology of different diseases, multiple clinical trials using PARP inhibitors have been performed, primarily for the treatment of cancer. One of the key findings was the discovery of a potent and specific beneficial effect of PARP inhibition in BRCA2-deficient tumours, due to the defect in homologous recombination repair (HRR) in the BRCA2-deficient cells [64, 65]. In fact, most of the clinical trials using PARP inhibitors for the treatment of different tumours that have been performed in the past or are currently being performed are based on the role of PARP-1 in the maintenance of genomic stability and integrity. Interestingly, preclinical and clinical trials have been successful not only in tumours with deficiencies in the double-stranded DNA repair, such as mutations in BRCA1 and BRCA2, but also in tumours that have no defects in the HRR [66–68].

Importantly, a protective effect of PARP inhibition has also been found in breast cancer through the attenuation of the NF-*κ*B-mediated signalling, independently of any defect in the homologous recombination DNA repair. PARP-1 inhibition may have a clear benefit in tumour treatment by limiting the rate of cell proliferation and activation of NF-*κ*B, leading to the suppression of both the inflammation and the expression of genes related to tumour progression [69]. These results suggest that PARP inhibitors might also be used in other inflammatory diseases in which NF-*κ*B has a critical role. Moreover, there is preclinical evidence showing that PARP-1 inhibitors would be beneficial in acute diseases, such as stroke, traumatic brain injury, circulatory shock, and acute myocardial infarction, as well as in chronic diseases such as chronic heart failure and asthma (reviewed by Curtin) [67].

Two important aspects of the PARP-1 inhibitors for their use as therapeutic target are the specificity and the toxicity and tolerability of the inhibitors. Regarding the specificity, most of the PARP-1 inhibitors that are currently in clinical trials also inhibit PARP-2 activity, as both PARP-1 and PARP-2 share significant sequence homology in their catalytic domains and these PARP inhibitors inhibit the PARP activity [40, 70]. The inhibition of both PARP-1 and PARP-2, rather than being a problem, may be beneficial in tumors associated with defective DNA repair, since PARP-2 is also related to the maintenance of the genomic stability. Regarding the toxicity and tolerability of the PARP-1 inhibitors, all the PARP-1 inhibitors which are or have been tested in clinical trial have not shown unacceptable side effect at the doses used. Alone or in combination with other drugs, PARP-1 inhibitors showed mild effects like nausea, vomiting, diarrhea, and fatigue [70–72].

Therefore, as PARP-1 inhibitors inhibit specifically the PARP enzymatic activity and are well tolerated for the patients, clinical trials are really promising and we can expect the use of PARP inhibitors for the treatment of different pathologies in the near future.

## 5. Conclusion

Intensive research on the pathology of RA has stimulated the introduction of novel approaches aimed at blocking the inflammatory cytokine pathways in the joint. The best example is the TNF blockade, which allows at least 20% improvement in approximately 70% of the patients. However, as many patients do not respond and remission is rarely achieved, there is a clear need for identifying novel therapeutic strategies. PARP-1, which has been shown to be an important regulator of inflammation in RA, is an interesting therapeutic target. The use of pharmacological PARP inhibitors, PARP-1-deficient mice, and siRNA technology have led to a better understanding of the role of PARP in arthritis and have indicated the potential therapeutic effects of PARP inhibition. Specifically, these studies have shown that PARP-1 is involved in the pathogenesis of RA and that PARP-1 inhibition may be used therapeutically in RA patients.

However, an important aspect of PARP biology that must be considered in the treatment of chronic diseases such as RA is the involvement of PARP in the maintenance of genomic stability. In RA, the long-term inhibition of PARP might increase the DNA mutation rate, thereby increasing the probability of tumour development. However, bearing in mind that the protective effect against arthritis of AIQ in the CIA model was mediated by a reduction of the autoimmune response [35], it is also possible to speculate that, in the future, PARP inhibitors will be developed that induce a strong diminution of the immune response, leading to the remission of RA and avoiding the need for chronic treatment.

## Figures and Tables

**Table 1:** Effect of PARP inhibition/deletion in experimental arthritis.

Treatment/genetic approach	Arthritis model	Major findings	Reference
Nicotinamide	Potassium peroxochromate-induced arthritis	Reduced arthritis severity. Reduction of phagocytic generation of reactive oxygen species.	[30]
Nicotinic acid amide	Collagen-induced arthritis	Reduced arthritis severity. Synergistic effect with thalidomide.	[31]
INH_2_BP	Collagen-induced arthritis	Reduced arthritis severity and incidence. Reduced neutrophil infiltration. Reduced O_2_ and N_2_ derived free radical production.	[33]
PJ34	Collagen-induced arthritis	Reduced arthritis severity and incidence. Reduced neutrophil infiltration. Reduced O_2_ and N_2_ derived free radical production.	[34]
AIQ	Collagen-induced arthritis	Reduced arthritis severity and incidence. Reduced inflammatory response. Reduced Th1-driven autoimmune response.	[35]
PARP-1 deficient mice	Arthritis induced by anti-collagen antibodies	Reduced arthritis severity. Reduced synovial inflammation and cartilage damage. Reduced IL-1*β* and MCP-1 expression.	[36]
GPI6150	Adjuvant-induced arthritis	Reduced paw edema. Reduced neutrophil infiltration.	[37]
